# The complete mitochondrial genome of Uruguayan native cattle (*Bos taurus*)

**DOI:** 10.1080/23802359.2019.1704639

**Published:** 2020-01-08

**Authors:** Shu-Jing Liu, Ji-Zhou Lv, Zhong-Yang Tan, Xing-Yi Ge

**Affiliations:** a College of Biology, Hunan University, Changsha, PR China;; b Institute of Animal Quarantine, Chinese Academy of Inspection and Quarantine, Beijing, PR China

**Keywords:** Uruguayan native cattle, *Bos taurus*, mitogenome

## Abstract

Uruguayan beef is one of the most popular products in the export market. In this study, we report the complete mitochondrial genome sequence of Uruguayan native cattle for the first time. The total mitochondrial genome sequence is 16,339 bp in length with the base composition of 33.4% for A, 27.2% for T, 26.0% for C, and 13.4% for G. The description of all genes is similar to the typical mitochondrial genomes of cattle. The annotated mitochondrial genome of Uruguayan native cattle would serve as an important genetic data set for further study.

The Uruguayan native cattle (*Bos taurus*) is a cattle breed mainly distributed in the Uruguayan plain (Vanderwaal et al. 2016). Beef production in Uruguay has been aimed at the export markets, which accounts for 78% of total beef production (Realini et al. 2009). At present, Uruguay is the sixth largest beef exporter in the world (Huertas et al. 2018). Beef consumption is growing rapidly. Increasing beef production and quality will help Uruguay to improve its competitiveness in the world beef market (Realini et al. 2009). At the same time, identification of beef types becomes important for importing countries and consumers. Based on gene sequence, random amplified polymorphic DNA (RAPD) and microsatellite (MS) markers have been used to evaluate pool-specific products which are quite valuable (Rincón et al. 2000; Armstrong et al. 2013). However, no available genetic sequence data of the cattle breed in Uruguay restricts the development of detection methods.

In this study, we sequenced and characterized the complete mitochondrial genome of Uruguayan native cattle. Muscle samples of Uruguayan native cattle were collected from Rocha (−34.480965°N; −54.33061°E). Genomic DNA was extracted from muscle tissues using TIANamp Genomic DNA Kit (TIANGEN BIOTECH, Beijing, China) following the manufactures instructions. The isolated DNA was stored at −80 °C in the Institute of Pathogen Biology and Immunology, College of Biology, Hunan University (Sample codes are HNUUCMITO180723). The mtDNA of Uruguayan native cattle is a circular double-stranded DNA molecule of 16,339 bp long (Genbank accession number: MN510465), including one control region (D-loop), 22 tRNA genes, 2 rRNA genes, and 13 protein-coding genes. The description of all genes is similar to the typical mitochondrial genomes of cattle (Hiendleder et al. 2008). The base composition of A, T, C, G accounted for 33.4%, 27.2%, 26.0%, and 13.4%, respectively. The length of 22 tRNA genes varies from 60 to 75 bp.

Mitochondrial DNA sequence of this studied breed was compared with published mitochondrial genomes of other cattle breeds from NCBI database. Maximum-likelihood based phylogenetic analysis was performed with the complete mitochondrial DNA sequences of each breed (Tamura and Nei 1993). In the phylogenetic tree, *Bos indicus, Bos taurus, Bubalus bubalis*, and *Bos taurus* cattle presented four separate main clades (Figure 1). The sister relationship of *B. indicus* and *B. taurus* was confirmed with high support values. Our results also strongly supported that Uruguayan native cattle was clustered with Korean breeds as sister species. In summary, we characterized the mitogenome sequence of Uruguayan native cattle in this study which would facilitate further studies on the genetics of Uruguayan native cattle population and beef typing methods development, and may help in prioritization and designing of the conservation plans.

**Figure 1.**
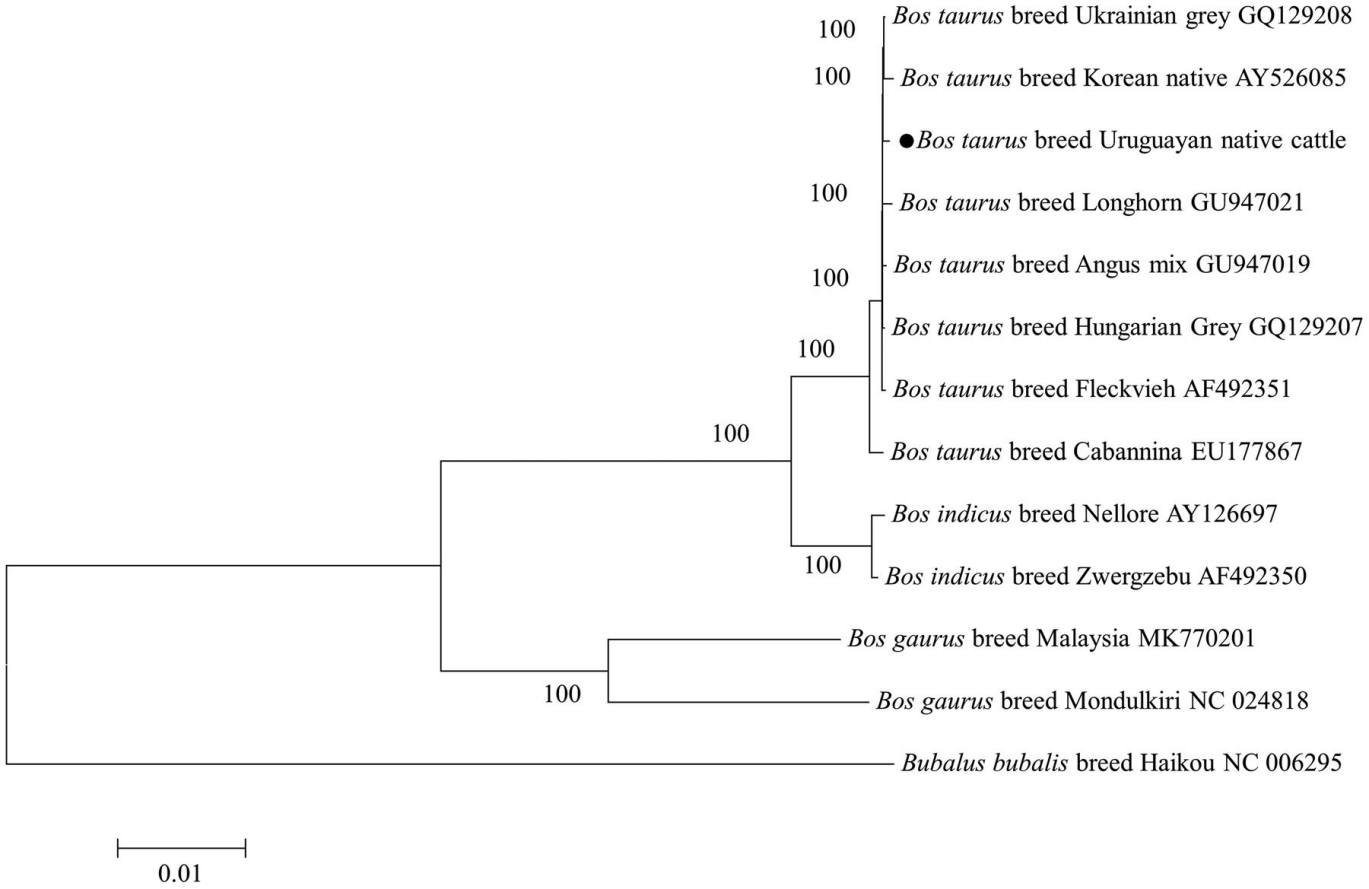
Phylogenetic analysis of cattle, based on the complete mitochondrial DNA sequences. Phylogenetic relationship between mtDNA sequences of Uruguayan native cattle and other available cattle mitogenomes were analyzed using maximum-likelihood method.
